# The N-terminal tail coordinates with carbohydrate recognition domain to mediate galectin-3 induced apoptosis in T cells

**DOI:** 10.18632/oncotarget.17760

**Published:** 2017-05-10

**Authors:** Huiting Xue, Lu Liu, Zihan Zhao, Zhongyu Zhang, Yuan Guan, Hairong Cheng, Yifa Zhou, Guihua Tai

**Affiliations:** ^1^ School of Life Sciences, Northeast Normal University, Changchun, China

**Keywords:** galectin-3, apoptosis, ERK, ROS, truncated protein

## Abstract

Galectin-3 is a galectin with a unique flexible N-terminal tail (NT) connected to the conserved carbohydrate recognition domain (CRD). Galectin-3 is associated with tumor immune tolerance and exhibits an ability to induce T cell apoptosis. We used Jurkat, Jurkat E6-1 and CEM T-cell lines and human peripheral blood mononuclear cells (PBMCs) to investigate the specific roles of the CRD and NT in inducing T cell apoptosis. Galectin-3 triggered sustained extracellular signal-regulated kinase (ERK) phosphorylation that induced apoptosis. ERK was situated upstream of caspase-9 and was independently activated by reactive oxygen species (ROS) and protein kinase C (PKC). The first twelve NT residues had no role in the apoptosis. Residues 13-68 were essential for activating ROS, but did not activate PKC. However, residues 69-110 were required for activation of PKC. An NT fragment and a NT-specific antibody antagonized the apoptosis triggered by full-length galectin-3 further supporting our findings. These findings indicate the CRD and NT play important roles during induction of T cell apoptosis, which suggests their potential as therapeutic targets for reversing cancer immune tolerance.

## INTRODUCTION

The mechanism of tumor escape from immune surveillance is usually associated with secretion of immune suppressive factors or pro-apoptotic stimulants that render immune cells either anergic or induce cell death [1]. Galectin-3 (Gal-3) is one such immune suppressive factor, which is highly expressed in malignant tumors [2, 3]. Gal-3 is one of the 15 galectin family members with an ability to bind β-galactosides through the conserved carbohydrate recognition domain (CRD) [4]. Gal-3 is a cytoplasmic protein that is secreted from cells via a non-classical secretory pathway [5]. Extracellular Gal-3 binds to glycan ligands on the cell surface and extracellular matrix or in bodily fluids and modifies cell-cell and cell-matrix adhesion, receptor turnover, and cell signaling [6, 7]. Elevated Gal-3 levels are observed in many malignant tumors [2], and it is a potential diagnostic and prognostic marker for some cancers [8]. Gal-3 promotes tumor growth and metastasis through multiple mechanisms [8], which include cellular transformation and invasion [6, 9, 10], angiogenesis [9, 10], and resistance to cancer therapeutics [11]. In addition, Gal-3 is implicated in tumor immune tolerance by impairing the function of tumor-infiltrating lymphocytes [12] and inducing T cell apoptosis [13]. Gal-3 is a target in the development of drugs against cancer because of its role in promoting cancer. A number of Gal-3 inhibitors have been developed, some of which have demonstrated promising anti-cancer characteristics [14, 16].

Gal-3-induced T cell apoptosis has been reported in several studies. Zubieta *et al* found that expression of Gal-3 correlated with apoptosis of tumor associated T cells in human melanomas [15]. In addition, serum Gal-3 obtained from patients with prostate cancer induced apoptosis in tumor-specific CD8^+^CD25^+^ T cells [16]. High expression of Gal-3 in human CD133^+^ lung adenocarcinoma cells induced apoptosis of CD8^+^ T cells [17]. A high dose injection of Gal-3 in a mouse tumor model resulted in inhibition of tumor-reactive T cells and promoted tumor growth [18]. Many studies have also shown that Gal-3 induced apoptosis in a variety of cells like the human T-leukemic cell lines, human peripheral blood mononuclear cells, activated primary human and mouse T cells and human tumor infiltrating T cells [13, 16–20]. Interestingly, the Gal-3 null cells (e.g. CEM, Jurkat and MOLT-4) were more sensitive than the Gal-3 positive cells (e.g. H9 and SKW6.4) [13]. Several receptors like CD7 and CD29 (β1 integrin) on MOLT-4 cells [13] and CD45 and CD71 on Jurkat E6-1 cells [19, 21] have been implicated in the Gal-3 activated apoptotic cascade. Although Gal-3 triggers apoptosis through cytochrome C release and caspase-3 activation [13], the details of all the signaling events in the apoptosis cascade are unknown.

Gal-3 is composed of the conserved CRD, and in contrast to other galectins, has a relatively long N-terminal tail (NT). Unlike the full-length Gal-3, the Gal-3C (CRD devoid of its NT) inhibited tumor growth and metastasis [22]. Also, Gal-3C did not activate neutrophils that produce interleukin 8 (IL-8) [23]. In addition, Gal-3C was unable to promote tube formation in angiogenesis, unlike the full length Gal-3 [24]. These data highlighted the importance of NT in Gal-3 function. While the CRD may be involved in glycan recognition, we postulated that NT maybe involved in inducing T cell apoptosis. Therefore, in this study, we studied key apoptotic signaling events that are triggered by Gal-3 in multiple T cell leukemia cell lines and peripheral blood mononuclear cells (PBMCs) and the roles of the CRD and NT domains by using different deletion constructs of Gal-3.

## RESULTS

### Gal-3 induced T cell apoptosis by activating ERK1/2

**Figure 1:**
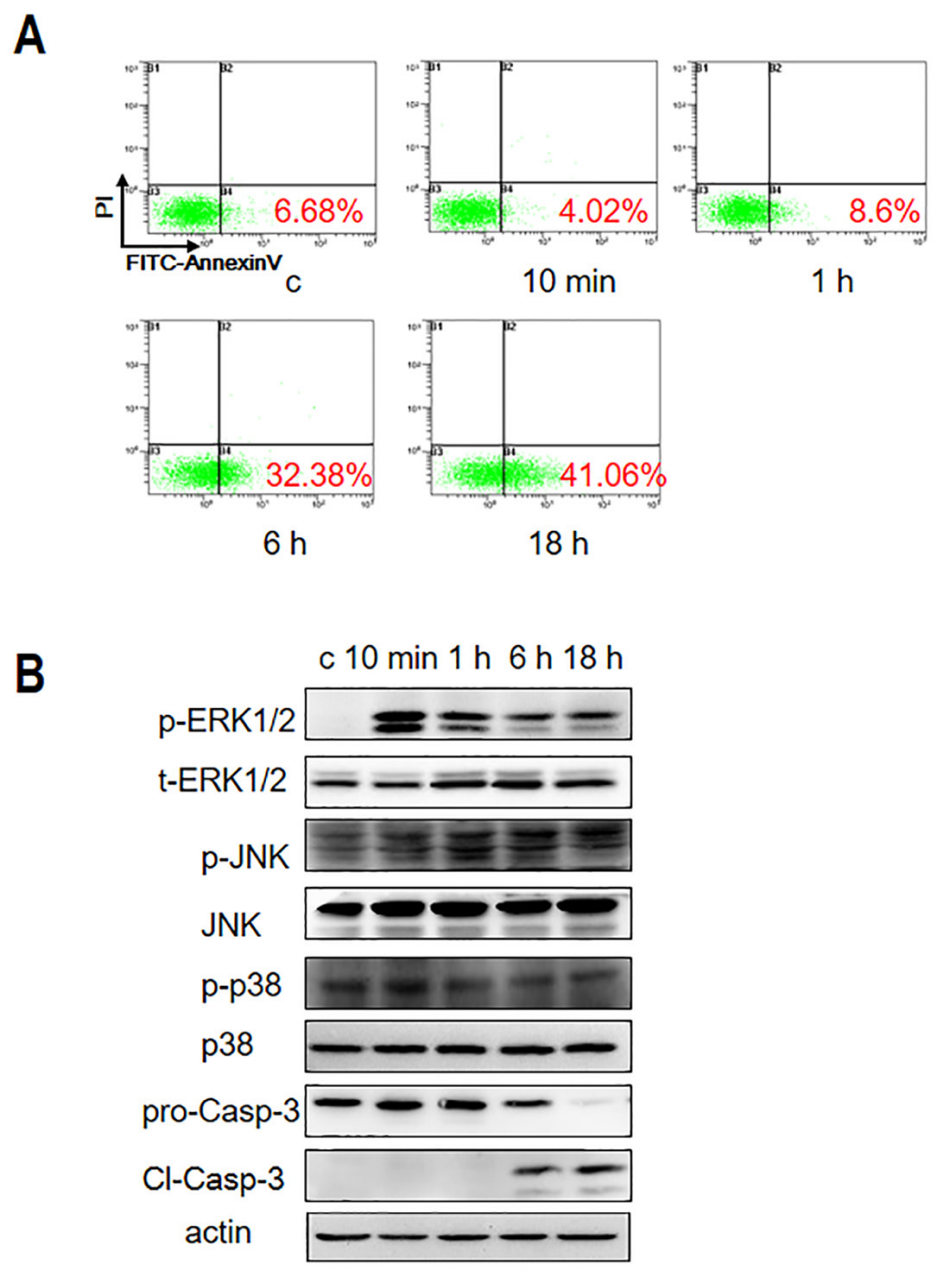
Gal-3 treatment induces Jurkat cell apoptosis **(A)** Jurkat cells were incubated with 2.5 μM Gal-3 for 10 min, 1 h, 6 h and 18 h and apoptosis was analyzed by PI/FITC-AnnexinV double staining and flow cytometry. **(B)** Gal-3-treated Jurkat cells were analyzed for the presence of phosphorylated and non-phosphorylated forms of ERK1/2, JNK and p38 MAPKs by western blotting. Also, full length (pro-Casp-3) and cleaved caspase-3 (Cl-Casp-3) were analyzed by western blotting.

To understand the mechanism by which Gal-3 induces apoptosis in T cells, we first analyzed apoptosis in the human leukemia T cell line, Jurkat cells by incubating them with 2.5 μM Gal-3 for 10 min, 1 h, 6 h and 18 h, respectively. Analysis by flow cytometry with PI/FITC-AnnexinV staining demonstrated that although apoptosis was low during the first hour, Gal-3 induced apoptosis in 32% and 41% Jurkat cells at 6 h and 18 h, respectively (Figure 1A). Consistent with the flow cytometry data, western blot analysis showed cleaved caspase-3 at 6 h and 18 h, but not at 1 h (Figure 1B). These data indicated that Gal-3 induced apoptosis in a time dependent manner.

To identify the signaling pathways involved in Gal-3-induced apoptosis, we investigated the role of MAPK family by analyzing the phosphorylation status of extracellular signal-regulated kinase 1 and 2 (ERK1/2), c-Jun amino terminal kinase (JNK), and p38, respectively. Western blot analysis demonstrated that phosphorylation of ERK occurred quickly after 10 min of incubation with Gal-3 followed by slight decline at 1 h and remained high at 6 h and 18 h (Figure 1B). In contrast, p-JNK and p-p38 levels were negligible over the same time course. These observations suggested that activated ERK1/2 plays a critical role in Gal-3-induced T cell apoptosis.

**Figure 2:**
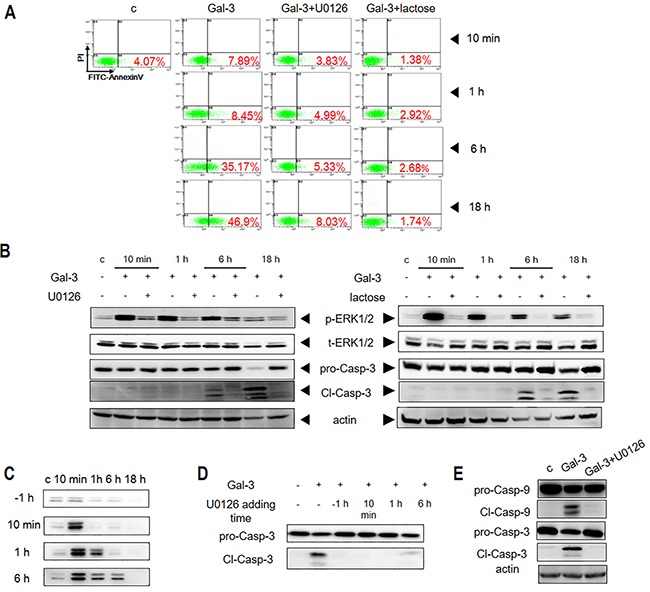
ERK phosphorylation is induced by Gal-3 in Jurkat cells **(A)** Jurkat cells were incubated with Gal-3 in the presence of U0126 or lactose for 10 min, 1 h, 6 h or 18 h and the apoptosis was measured by flow cytometry after PI/FITC-Annexin V double staining or **(B)** western blotting. **(C)** Jurkat cells were incubated with Gal-3 for 10 min, 1 h, 6 h or 18 h in the presence of U0126, which was added 1 h before (−1h) or 10 min, 1 h or 6 h after addition of Gal-3. P-ERK1/2 was then assessed by western blotting. **(D)** Jurkat cells were incubated with Gal-3 for 18 h in the presence of U0126 as described in C and the cleavage of caspase-3 was determined by western blotting. **(E)** Jurkat cells were treated with Gal-3 for 18 h in the presence or absence of U0126 and the cleavage of caspase-9 and caspase-3 were analyzed by western blotting. The data are representative of three independent experiments.

To determine if ERK activation was critical for Gal-3-induced apoptosis, we treated the Jurkat cells with the ERK-specific inhibitor U0126 in presence of Gal-3 and observed inhibition of ERK phosphorylation and apoptosis (Figure 2A-2B). In contrast, SP600125 and SB203580, the specific inhibitors of JNK and p38, respectively, did not inhibit apoptosis though they suppressed basal levels of p-JNK and p-P38 (Supplementary Figure 1A-1B). These data demonstrated that ERK phosphorylation was required for Gal-3-induced apoptosis. To further evaluate the involvement of ERK phosphorylation, we further showed that lactose, the Gal-3 CRD competitive inhibitor [13, 25] completely inhibited ERK phosphorylation and apoptosis when Jurkat cells were treated with Gal-3 (Figure 2A-2B). Overall, our results demonstrated that ERK phosphorylation was essential for Gal-3-induced T cell apoptosis.

We also tested if phosphatidylinositol-3 kinase (PI3K), AKT and c-Raf were activated by Gal-3 and observed that although they were phosphorylated (Supplementary Figure 1C and 1F), their inhibition by specific inhibitors (LY294002 for PI3K, MK2206 for Akt, and GW5074 for Raf) did not inhibit Gal-3-induced apoptosis (Supplementary Figure 1D-1F). This suggested that PI3K, AKT and c-Raf were not involved in apoptotic signaling by Gal-3. We also demonstrated that the small GTPase, Ras, was not involved in apoptotic signaling as S-Farnesylthiosalicylic acid (FTS, Ras inhibitor) did not inhibit Gal-3-induced apoptosis (Supplementary Figure 1G).

### Sustained ERK phosphorylation is required for Gal-3-induced apoptosis

We observed that the Gal-3-induced ERK phosphorylation endured for an extended period of time. Therefore, we investigated if sustained ERK phosphorylation by Gal-3 was necessary for apoptosis by inhibiting ERK with U0126 inhibitor at different time points (Figure 2C). We observed that ERK phosphorylation was completely inhibited when U0126 was added 1 h prior to Gal-3 induction, whereas addition of U0126 at 10 min, 1 h or 6 h resulted in varied inhibition of p-ERK (Figure 2C). Interestingly, cleaved caspase-3 was absent when the inhibitor was added at 10 min and 1 h, although ERK phosphorylation levels at these time points was similar to those in the absence of inhibitor (Figure 2D). Thus, early phosphorylation (≤1 h) was insufficient to induce apoptosis, whereas late phosphorylation (>1 h) was required to complete the apoptotic process. Consistent with this conclusion, we noted that fractional apoptosis was observed when phosphorylation lasted for 6 h, and full apoptosis was observed when phosphorylation lasted for 18 h (i.e. without inhibitor) (Figure 2D).

### ERK phosphorylation is upstream of caspase-9 in Gal-3 induced apoptosis

We then explored if ERK regulated caspase-9, an initiator caspase, which is upstream of executioner or effector caspases like caspase-3. We observed that the ERK inhibitor U0126 inhibited activation of caspase-9 in Jurkat cells treated with Gal-3 (Figure 2E). These results indicated that ERK was upstream of caspase-9.

### Reactive oxygen species regulates ERK phosphorylation during Gal-3 induced apoptosis

**Figure 3:**
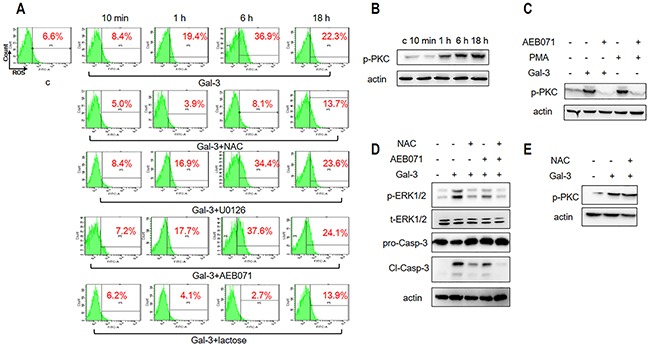
ROS and PKC activation is necessary for Gal-3-induced apoptosis **(A)** Jurkat cells were incubated with Gal-3 for 10 min, 1 h, 6 h and 18 h in the absence or presence of NAC, U0126, AEB071, or lactose and analyzed by flow cytometry for ROS as described in the Materials and Methods. **(B)** Jurkat cells were treated with Gal-3 for 10 min, 1 h, 6 h and 18 h and analyzed for p-PKC by western blotting. **(C)** Jurkat cells were incubated with Gal-3 or PMA in the absence or presence of AEB071 for 18 h and analyzed for p-PKC by western blotting. **(D)** Jurkat cells were treated with Gal-3 for 18 h in the absence or presence of AEB071 and NAC and were analyzed for p-ERK and cleaved caspase-3. **(E)** Jurkat cells were incubated with Gal-3 in the absence or presence of NAC for 18 h. p-PKC was analyzed by western blotting. The data are representative of three independent experiments.

Reactive oxygen species (ROS) have been implicated in both apoptotic and non-apoptotic processes [26–28]. Therefore, we explored if ROS regulated Gal-3-induced ERK phosphorylation and cellular apoptosis by treating the Jurkat cells with 2.5 μM Gal-3 for 10 min, 1 h, 6 h or 18 h and assessed the cellular ROS levels by flow cytometry using the fluorescent probe H_2_DCFDA. We observed that cellular ROS remained low at 10 min after Gal-3 treatment, but increased significantly at 1 h and peaked at 6 h followed by subsequent lowering thereafter (Figure 3A). This indicated that Gal-3 induced apoptosis involved ROS production.

To determine if ROS directly participated in inducing apoptosis, we treated Jurkat cells with either N-Acetyl Cysteine (NAC) that scavenges cellular ROS or lactose that inhibits Gal-3-induced apoptosis. Our results showed that NAC, which inhibits ROS production (Figure 3A), also inhibited cellular apoptosis (Figure 3D). Conversely, lactose that inhibited Gal-3 induced apoptosis (Figure 2A-2B) also suppressed ROS production (Figure 3A). These data suggested that ROS is central to Gal-3-induced apoptosis.

Next, we investigated the sequential order of signaling activation between ROS and ERK activation by using NAC and U0126. Our results showed that whereas NAC inhibited ERK phosphorylation (Figure 3D), U0126 did not inhibit ROS production (Figure 3A). Thus, we concluded that ROS production was upstream of ERK activation during Gal-3 induction of apoptosis.

### Activation of Protein Kinase C in combination with ROS regulates ERK phosphorylation during Gal-3 induced apoptosis

Members of the protein kinase C (PKC) family play key roles in signal transduction by promoting serine/threonine phosphorylation in cell metabolism, proliferation, apoptosis and differentiation [29]. Therefore we investigated the status of phosphorylated PKC (p-PKC) when cells were treated with Gal-3 and observed that p-PKC levels increased over time (Figure 3B). To determine if PKC activation was related to ERK phosphorylation, we treated cells with the PKC inhibitor AEB071 that inhibits PKC phosphorylation induced by PMA (4β-phorbol 12-myristate 13-acetate), a specific activator of PKC (Figure 3C). We observed that the PKC inhibitor AEB071 inhibited ERK phosphorylation and cellular apoptosis in addition to PKC phosphorylation (Figure 3C-3D). This suggested that PKC probably activated ERK either directly or indirectly. We further observed that combined treatment of cells with both AEB071 and NAC resulted in highly reduced levels of both ERK phosphorylation and cellular apoptosis compared to treatment with either drugs alone (Figure 3D).

### PKC and ROS pathways independently and synergistically regulate ERK phosphorylation and apoptosis

Since both PKC and ROS lead to ERK phosphorylation, we investigated if they were part of the same pathway or not. We observed that treatment of PKC inhibitor AEB071 had no effect on ROS production (Figure 3A), indicating that PKC activation was independent of ROS. Conversely, treatment with ROS inhibitor NAC did not inhibit PKC phosphorylation upon Gal-3 stimulation (Figure 3E) suggesting that ROS induction was not related to PKC activation. Finally, neither NAC nor AEB071 inhibited cellular apoptosis maximally (Figure 3D). These results suggested that PKC and ROS pathways contribute independently and synergistically to ERK phosphorylation and apoptosis.

### Apoptotic pathways in Jurkat E6-1, CEM and PBMCs are similar to Jurkat cells

**Figure 4:**
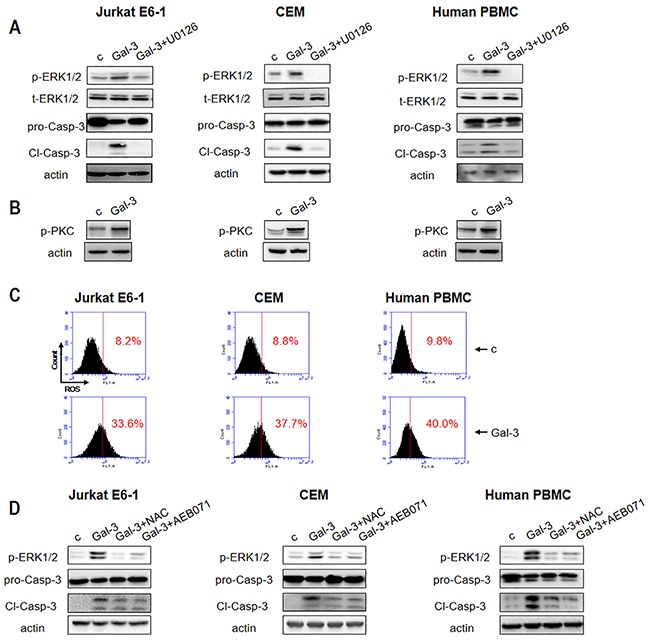
Gal-3 triggers apoptotic signaling in different T leukemia cell lines and PBMCs **(A)** Jurkat E6-1, CEM and human PBMC were incubated with Gal-3 in the presence or absence of U0126 for 18 h followed by analysis for p-ERK1/2 and cleaved caspase-3 and **(B)** p-PKC by western blotting. **(C)** Jurkat E6-1, CEM and human PBMCs were incubated with Gal-3 for 6 h and ROS production was detected by flow cytometry. **(D)** Jurkat E6-1, CEM and human PBMCs were incubated with Gal-3 for 18 h in the absence or presence of NAC and AEB071 followed by analysis of p-ERK1/2 and cleaved caspase-3 by western blotting.

Having shown that ERK and PKC phosphorylation in combination with ROS was central to apoptosis in Jurkat cells treated with Gal-3, we investigated if similar pathways were activated in two other human T-leukemic cell lines (Jurkat E6-1 and CEM) and human peripheral blood mononuclear cells (PBMCs). As shown in Figure 4A, Gal-3 induced ERK phosphorylation and caspase-3 activation in the Jurkat E6-1, CEM and the PBMCs, which could be effectively inhibited by ERK inhibitor U0126. Also, Gal-3 enhanced phosphorylation of PKC (Figure 4B) and ROS production (Figure 4C). Further, AEB071 (PKC inhibitor) and NAC (ROS scavenger) inhibited both ERK phosphorylation and caspase-3 activation (Figure 4D). These data indicated that phosphorylation of ERK and PKC, and ROS production was central to Gal-3 induced apoptosis in general.

### N-terminal tail of Gal-3 is necessary for induction of apoptosis

**Figure 5:**
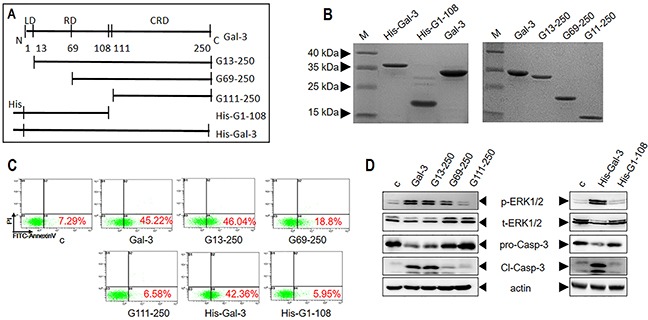
Characterization of apoptotic signaling efficiency of truncated variants of Gal-3 **(A)** Schematic details of Gal-3 and its variants. **(B)** SDS-PAGE analysis of Gal-3 and its variants. **(C-D)** Jurkat cells were treated with 2.5 μM Gal-3 or its variants for 18 h and (C) cellular apoptosis was assessed by flow cytometry and (D) signaling was assessed by western blot analysis of p-ERK1/2 and cleaved caspase-3.

Gal-3 is composed of a conserved CRD (135 residues) domain and an N-terminal tail (NT, ∼110 residues) comprising a leader domain (LD, initial 12 residues) and a collagen-like internal repeating domain (RD) [8]. Therefore, to assess the association of each of these domains with apoptotic signaling pathways, we first prepared various truncated forms of Gal-3 (Figure 5A-5B). The full length lectin and NT-truncated forms (G13-250, G69-250, and G111-250) were prepared as previously reported [30], whereas the His-tagged Gal-3 (His-Gal-3) and its CRD-truncated variant (His-G1-108) were prepared as described in the Materials and Methods section.

Then, we investigated the ability of the Gal-3 variants to induce apoptosis in comparison to the wild type Gal-3 by treating Jurkat cells with 2.5 μM of each of the different Gal-3 variants (Figure 5C-5D). Our results showed that both His-G1-108 (without CRD) and G111-250 (without NT) could not induce apoptosis (Figure 5C) suggesting that both domains were necessary for apoptosis. However, the positive control His-Gal-3 induced apoptosis as efficiently as untagged Gal-3 (Figure 5C). Further, we observed that G13-250 (without LD) exhibited maximal activity as full length Gal-3, whereas G69-250 (devoid of LD plus part of RD) showed reduced apoptosis (19% compared to 45% in positive control) suggesting that the collagen-like internal repeating domain (RD) of the N-terminal tail was necessary for maximal apoptosis (Figure 5C).

### N-terminal tail of Gal-3 activates ROS during apoptotic signaling

**Figure 6:**
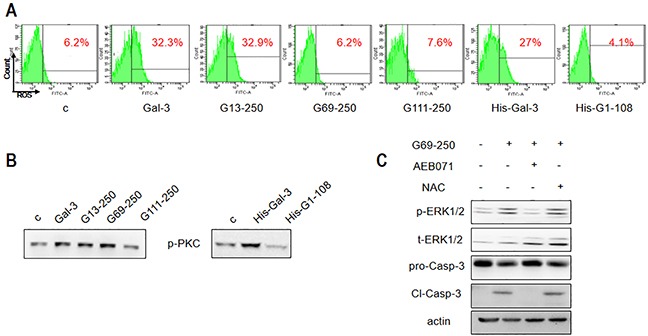
Differential apoptotic signaling induced by Gal-3 and its truncated variants Jurkat cells were treated with 2.5 μM Gal-3 or its variants for 18 h, and **(A)** ROS production was assessed by flow cytometry and **(B)** p-PKC was detected by western blotting. **(C)** Jurkat cells were treated with 2.5 μM G69-250 in the absence or presence of AEB071 or NAC for 18 h and analyzed for p-ERK1/2 and cleaved caspase-3. The data are representative of three independent experiments.

We further investigated the ability of the truncated Gal-3 variants to induce ERK and PKC phosphorylation and ROS production. We observed that the Gal-3 variants, His-G1-108 and G111-250 that were unable to induce apoptosis neither activated ERK, PKC nor generated ROS (Figure 5D and Figure 6). The G13-250 demonstrated full activation of ERK and PKC, as well as enhanced ROS in accordance with its ability to maximally induce apoptosis (Figure 5D and Figure 6). Interestingly, the G69-250 variant showed partial activation of ERK (Figure 5D) and inhibition of ROS generation (Figure 6A), but fully induced PKC phosphorylation (Figure 6B). This suggested G69-250 activates the PKC-ERK pathway, but not the ROS-ERK pathway. This was supported by our data that whereas the PKC inhibitor AEB071 inhibited G69-250-induced ERK and caspase-3 activation fully, the ROS scavenger NAC had no effect (Figure 6C). Further, our data that G13-250 variant could fully activate ROS, but the G69-250 variant was unable to induce ROS demonstrated that a short sequence within the RD (residues 13 to 68) was required for ROS induction. In addition, our observation that the G69-250 variant, but not the G111-250 variant, fully activated PKC indicates that residues 69 to 110 were required for PKC induction.

### The antagonists of NT and CRD suppress Gal-3-induced apoptosis

**Figure 7:**
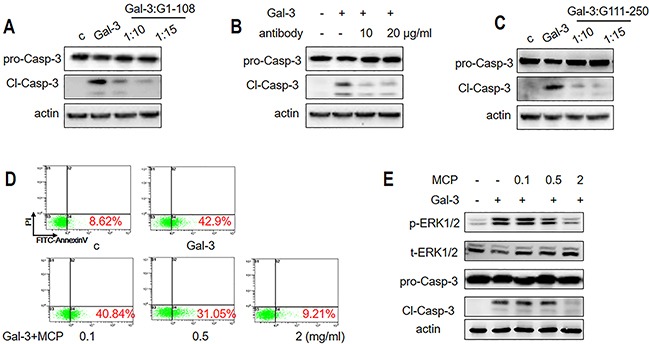
Gal-3-induced T cell apoptosis is inhibited by NT or CRD inhibitors Jurkat cells were incubated with 1 μM Gal-3 for 18 h in the presence or absence of **(A)** 10 or 15 μM G1-108 variant, **(B)** 10 or 20 μg/ml A3A12 antibody or **(C)** 10 or 15 μM G111-250 variant. Caspase-3 cleavage was assessed by western blot. **(D-E)** Jurkat cells were treated with 2.5 μM Gal-3 in the absence or presence of 0.1, 0.5 and 2 mg/ml MCP for 18 h. The apoptotic rate was measured by flow cytometry (D), and p-ERK1/2 and cleaved caspase-3 were determined by western blotting (E). The data are representative of three independent experiments.

To further verify the role of NT, we investigated if the G1-108 variant (NT fragment alone) and a monoclonal antibody A3A12 (Abcam, Cambridge, UK), which recognizes the first 45 residues in NT [31] antagonized apoptosis induced by the full length Gal-3 protein. The results showed that G1-108 exhibited inhibition at the molar ratio of 1:10 and 1:15 (Gal-3: G1-108) (Figure 7A), whereas the A3A12 antibody inhibited apoptosis at a concentration of 10 μg/ml (Figure 7B). These data further demonstrated that NT was critical for induction of T cell apoptosis by Gal-3.

The role of CRD was also confirmed by the competitive inhibition of the CRD fragment, variant G111-250, which markedly decreased apoptosis at a molar ratio of 1:10 and 1:15 (Gal-3: G111-250) (Figure 7C). We further tested the role of the CRD by testing if MCP, a pectin-derived polysaccharide that inhibits tumor growth and metastasis by targeting the Gal-3 CRD [32] inhibited Gal-3-induced T cell apoptosis. When Jurkat cells were treated with Gal-3 in the presence of 0, 0.1, 0.5 and 2 mg/ml MCP, the apoptosis rate was reduced from 43% to 41%, 31%, and 9.2%, respectively (Figure 7D). Western blot analysis showed that cleaved caspase activity was inhibited confirming the inhibition of apoptosis (Figure 7E). In addition, ERK phosphorylation was significantly inhibited by 2 mg/ml MCP (Figure 7E), consistent with the role of ERK in Gal-3 induced apoptosis.

### CD45 mediates activation of both PKC and ROS during Gal-3 induced apoptosis

**Figure 8:**
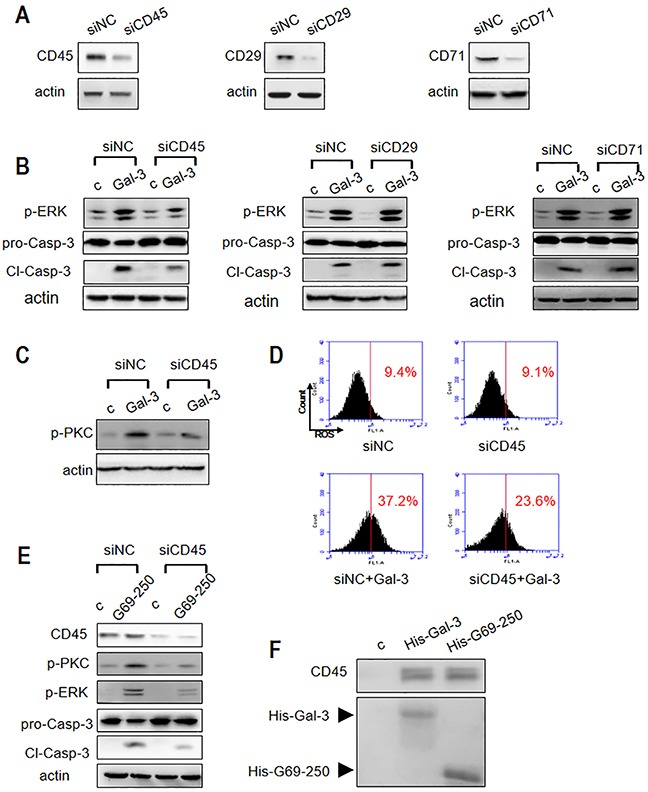
Roles of T cell surface receptor molecules in Gal-3-triggered T cell apoptosis **(A)** Jurkat cells were transfected with siRNAs to CD45, CD29 or CD71 and compared with the negative control siRNA (siNC). The knockdown efficiency of CD45, CD29 and CD71 was determined by western blotting. **(B)** Jurkat cells from (A) were incubated with or without 2.5 μM Gal-3 (c refers to control). P-ERK1/2 and cleaved caspase-3 was determined by western blotting. **(C-D)** Control or CD45 knocked-down Jurkat cells were incubated with or without 2.5 μM Gal-3 and p-PKC was determined by western blotting (C) and ROS production was analyzed by flow cytometry (D). **(E)** Control or CD45 knocked-down Jurkat cells were incubated with or without 2.5 μM G69-250 variant and then analyzed for CD45, p-ERK, p-PKC and cleaved caspase-3. **(F)** Jurkat cell lysate was incubated with His-Gal-3- or His-G69-250-immobilized Ni-NTA beads. The upper panel shows CD45 determined by western blotting and the lower panel shows SDS-PAGE analysis of His-Gal-3 or His-G69-250 on the beads.

Next, we analyzed the T cell surface proteins that transmit the apoptotic signal. Specifically, we focused on CD29, CD71 and CD45, which have been implicated in Gal-3 triggered T cell death [13, 19, 21]. First, we knocked down these proteins by small interfering RNA (siRNA) and analyzed their effect on Gal-3-induced Jurkat cell apoptosis. As shown in Figure 8A, the expression of CD29 and CD71 was significantly downregulated by respective siRNA treatments. We observed that knockdown of CD29 and CD71 did not affect Gal-3-induced ERK phosphorylation and caspase-3 activation (Figure 8B). In contrast, knockdown of CD45 (nearly 65%; Figure 8A, lanes siCD45 with siNC) markedly decreased Gal-3-induced ERK phosphorylation and caspase-3 activation (Figure 8B). This demonstrated that CD45 was involved in Gal-3-induced Jurkat cell apoptosis. Further, we observed that both p-PKC expression (Figure 8C) and ROS production (Figure 8D) were decreased in CD45 knockdown cells suggesting that CD45 mediated activation of both pathways. The involvement of CD45 in PKC pathway was further demonstrated with the G69-250 variant that activates PKC, but not ROS. As shown in Figure 8E, knock down of CD45 inhibited caspase-3 activation, and phosphorylation of both ERK and PKC. Also, both the full-length Gal-3 and G69-250 pulled down CD45 demonstrating that they directly interacted at the T cell surface (Figure 8F).

## DISCUSSION

Gal-3, a member of the galectin family triggers apoptosis through cytochrome C release and subsequent caspase-3 activation in T cells [13]. Apart from Gal-3, other galectins like Gal-1, -2, -4, -8 and -9 also trigger apoptosis in T cells by different mechanisms [33–37]. Gal-1 induces T cell apoptosis via the JNK/c-Jun/AP-1 pathway [33], whereas Gal-8 induces T cell apoptosis by activating ERK through the PLD/PA pathway [36]. Our study demonstrates that Gal-3 induces apoptosis by activating ERK and PKC and ROS. Thereby, it differs from Gal-1 since it does not activate JNK like Gal-1. Also, other galectins do not induce ROS during T cell apoptosis as demonstrated by Gal-3.

We also showed that PI3K, AKT and c-Raf were not involved in Gal-3 induced apoptosis. In many instances, the small GTPase Ras is an important signaling molecule in cell survival/apoptosis [48, 49]. Cytoplasmic Gal-3 has been shown to preferentially bind K-Ras and activate Ras signaling [38]. However, we demonstrated that Ras was not involved in Gal-3 induced apoptosis as demonstrated by the Ras inhibitor, S-Farnesylthiosalicylic acid (FTS) that inhibits PI3K phosphorylation [39]. Furthermore, Ras and Raf are common upstream molecules of ERK [40, 41]. Therefore, our data demonstrated that ERK activation induced by Gal-3 was not via Ras and Raf.

Previously, Gal-3 has been reported to induce mitochondrial pathway of apoptosis via cytochrome C release, whereas casapse-8 is not involved [13]. Here, we demonstrated that caspase-9 was activated suggesting that the cytochrome C/Apaf-1/caspase-9 acted as an apoptosome to amplify the caspase cascade [42] that finally activated the effector casapse-3 to induce apoptosis. However, it must be noted that alternate pathways exist for caspase-9 cleavage by other caspases and alternative pathways not involving Apaf-1 and cytochrome C [43, 44].

Four cell surface glycoproteins including CD7, CD29, CD45 and CD71 have been reported to be potential death receptors on T cells [13, 19, 21]. Here we showed that CD45 was required for activating both the PKC and ROS pathways. It was not surprising that reduced CD29 expression did not affect apoptosis because previously CD29^null^ Jurkat cells had been shown to be susceptible to Gal-3-triggered cell death [19], although anti-CD29 antibody inhibited apoptosis in MOLT-4 cells [13]. Further, knocking down CD71 did not suppress Gal-3 induced apoptosis similar to that observed in Jurkat E6-1 cells [19]. In fact, redistribution of CD71 was observed during Gal-3-triggered cell death [19]. We also observed reduced CD71 expression during Gal-3 induced apoptosis (data not shown), which may explain why CD71 knockdown did not inhibit apoptosis. However, the functional role of CD71 in this process remains to be determined.

Although the function of the Gal-3 CRD is well known, the role of NT had not been realized previously. Here, we demonstrate that NT is essential to induce apoptosis. Notably, we showed that different segments of NT played specific roles in this process. It has been proposed previously that although Gal-3 exists primarily as a monomer in solution, it associates as a dimer or pentamer via the flexible NT upon binding multivalent carbohydrates [45]. However, in this case, oligomerization could not explain the functional differences exerted by different segments of Gal-3. However, NT interacts with other proteins [46] and it is plausible that NT sequences 13-68 and 69-110 bind to different proteins on the cell surface, thereby activating different apoptotic pathways. This speculation is not in conflict with our observation that both full-length Gal-3 and the G69-250 variant interacted with CD45. In this regard, CD45 alone may not be sufficient to initiate one or both pathways. Other cell surface proteins such as TCR-CD3, CD4/CD8, CD26, CD28, CD58, and CD100, which complex with CD45 on the T- cell surface [47] may also be involved. However, the roles of other cell surface receptors in the Gal-3 induced apoptotic pathway remains to be established.

Our finding that the RD (residues 13-110) was required for Gal-3 induced apoptosis and not LD (residues 1-12) is consistent with our previous study that showed that RD (but not the LD) was required for Gal-3 endocytotic trafficking to the late endosome/lysosome [30]. In this regard, it is interesting to investigate whether Gal-3-induced endocytosis was relevant to apoptosis. Fukumori *et al* reported that deletion of the first 62 NT residues negated the pro-apoptotic effects in MOLT-4 cells [13]. We found that deletion of the first 68 residues maintained partial pro-apoptotic function in Jurkat cells. One possible reason for this discrepancy may be the use of different cell types in the two studies.

Since Gal-3 promotes cancer growth and metastasis, extensive studies have focused on the development of Gal-3 inhibitors. Among these, the pectic-derived polysaccharides exhibit relatively low toxicity compared to others [32]. Previously, MCP demonstrated inhibition of Gal-3 and the ability to inhibit cancer cell adhesion, homotypic aggregation, invasion, angiogenesis, and sensitization of neoplastic cells to apoptosis induced by chemotherapeutic agents, and correction of the impaired function of tumor-infiltrating lymphocytes [32]. In this study, we demonstrated that MCP potently inhibited Gal-3-induced T cell apoptosis suggesting its potential to treat tumor induced immune dysfunction via Gal-3. In this regard, targeting Gal-3 might restore T cell function and counteract tumor immune tolerance. Also, our findings that the NT fragments and the NT antibody antagonize Gal-3 induced apoptosis suggest greater scope for Gal-3 inhibitors in anticancer drug research. Importantly, the inhibitors targeting NT will be selective because NT is present only in Gal-3 but not in other galectin family members. Therefore, the selective inhibition of Gal-3 may avoid adverse effects associated with the inhibitors targeting CRD.

**Figure 9:**
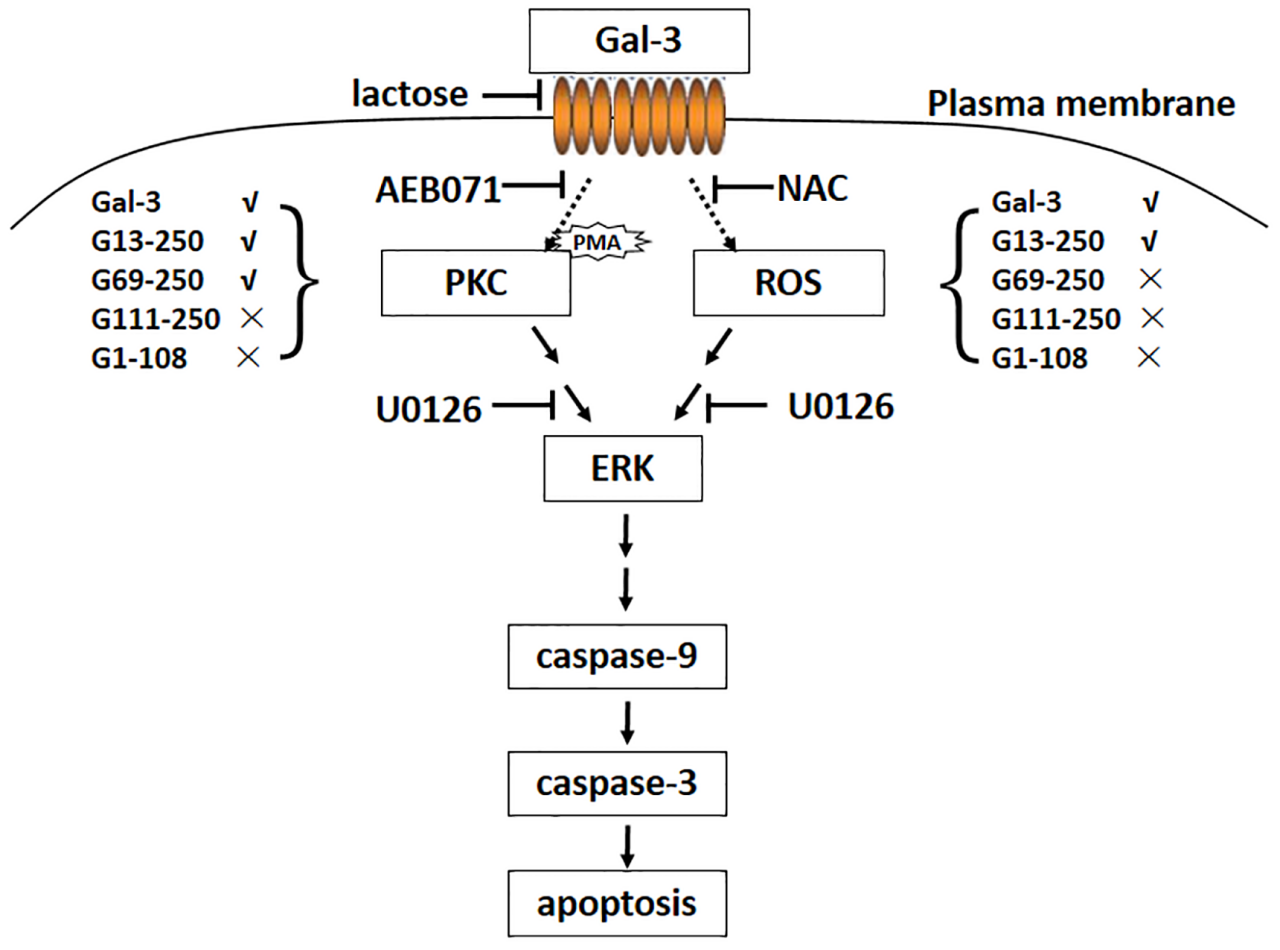
Model for Gal-3-induced T cell apoptosis The interaction of Gal-3 with cell surface death receptors activates ERK via ROS and PKC. The death signal is then conveyed from ERK to caspase-9 that activates caspase-3 and the downstream pathways that ultimately result in cell apoptosis. As with full length Gal-3, the truncated G13-250 variant can activate both pathways, whereas the G69-250 variant can only activate the PKC pathway. The G111-250 and G1-108 variants were unable to activate both pathways.

In conclusion, we demonstrated that Gal-3 induces apoptosis by activating ERK via independent and synergistic activation of PKC and ROS by interacting with the CD45 T cell receptor. Further, we demonstrated that the N-terminal tail of Gal-3, especially residues 13-68 had a significant role in activation of the apoptotic pathways. Based on these results, we propose a model for Gal-3-induced T cell apoptosis as illustrated in Figure 9.

## MATERIALS AND METHODS

### Cell culture

Human leukemic T cell lines Jurkat, Jurkat E6-1 (Jurkat clone E6-1) and CCRF-CEM that were obtained from ATCC were cultured in RPMI 1640 medium (Gibco, NY, USA) supplemented with 1% penicillin-streptomycin (PAN Biotech, Aidenbach, Germany) and 10% fetal bovine serum (Gibco, NY, USA) in a humidified 5% CO_2_ incubator at 37°C.

### Preparation of human PBMCs

PBMCs were obtained from whole blood of healthy donors, recruited from the Northeast Normal University Clinic, using Lymphoprep^TM^ (Alere Technologies AS, Oslo, Norway) density gradient separation solution. Briefly, 30 ml of whole blood were diluted with an equal volume of 0.9% NaCl and layered over Lymphoprep solution (6 ml of the whole blood diluents and 3 ml of Lymphoprep solution per centrifuge tube). After centrifugation at 800 g for 30 min, the interphase layer containing PBMCs was collected and washed thrice with RPMI 1640. The PBMCs were cultured in RPMI 1640 medium supplemented with 10% fetal bovine serum and 1% penicillin-streptomycin.

### Preparation of Gal-3 and its variants

Recombinant human Gal-3 and its truncated forms (G13-250, G69-250 and G111-250) were prepared as previously reported [30]. To generate His-tagged full-length Gal-3 (His-Gal-3), G69-250 (His-G69-250, residues 69-250) and G1-108 (His-G1-108, residues 1-108) proteins, plasmid pOTBT (Sanying Biotechnology, Wuhan, China) harboring the full sequence of human Gal-3 was used as a PCR template to amplify the cDNAs of Gal-3, G69-250 and G1-108. The following primers were used for PCR: Gal-3 cDNA, forward primer 5′-agccatatggcagacaatttttc-3′ and reverse primer 5′-tgggatccagattatatcatggtat-3′; G69-250 cDNA, forward primer 5′-taacatatggcttatcccggagca-3′ and reverse primer 5′-tgggatccagattatatcatggtat-3′; G1-108 cDNA, forward primer 5′-atacatatggcagacaatttttcgct-3′ and reverse primer 5′-atggatccttagccataggggcca-3′. The PCR protocol entailed incubation at 94°C for 2 min, followed by 30 cycles of 94°C for 45 s, 60°C for 45 s, and 72°C for 2 min, and a final extension step at 72°C for 10 min. The cDNAs were cloned into pET-28a (+) between NdeI and BamHI restriction sites and the resulting constructs were transformed into *E. coli* BL21 (DE3) cells to express the fusion protein by induction with 0.25 μM IPTG for 12-16 h at 25°C. The proteins were extracted and purified by Nickel beads (Qiagen, GmbH, Hilden, Germany) according to the manufacturer's instructions. The purity of the proteins was analyzed by SDS-PAGE. The untagged G1-108 was generated by removing the His-tag from the His-G1-108 protein by digestion with the enzyme thrombin.

### Cell apoptosis assay

Jurkat cells (2×10^6^ cells/well) in serum free medium were seeded into 12-well plates and co-cultured with 2.5 μM Gal-3 or other truncated proteins for 10 min, 1 h, 6 h and 18 h. Whenever inhibitors were used (10 μM U0126, 10 μM AEB071, 40 mM NAC, 5 μM SP600125, 5 μM SB203580, 50 μM LY294002, 10 μM MK2206, 1 μM FTS or 50 nM GW5074), they were added to cells 1 h prior to stimulation with Gal-3 or other truncated proteins. The Gal-3 antagonists, lactose (2 mg/ml) or MCP (0.1, 0.5, 2 mg/ml) were added to cells at the same time as Gal-3, whenever used. When cells were stimulated with PMA (100 nM), it was added instead of Gal-3. For the competition analyses, the apoptosis assay was performed with 1 μM Gal-3 in presence of either 10 μM or 15 μM G111-250 or G1-108 variants or with 10-20 μg/ml A3A12 antibody (Abcam, Cambridge, UK) added together. Cell apoptosis was analyzed by flow cytometry (PI/FITC-AnnexinV double staining) and/or western blotting for the active form of caspase-3 (cleaved caspase) as described in the following section.

For the Jurkat E6-1 and CCRF-CEM cells, 2×10^6^ cells were incubated with 5 μM Gal-3 for 18 h. For the human PBMCs, 1×10^7^ cells were incubated with 10 μM Gal-3 for 18 h. The cells were then analyzed by flow cytometry for apoptosis (PI/FITC-AnnexinV double staining) or ROS or analyzed by western blotting for status of ERK, PKC and caspase-3 as described.

U0126 was purchased from Cell Signaling Technology (Beverly, MA, USA). AEB071, GW5074, SB203580, LY294002, MK2206 and FTS were from Selleck Chemicals (Houston, TX, USA). SP600125 was from Sigma Aldrich (St. Louis, MO, USA). PMA, N-acetylcysteine (NAC) and Reactive Oxygen Species (ROS) Kit were purchased from Beyotime (Shanghai, China). PI/FITC-Annexin V Apoptosis Detection Kit (KGA 107) was from KeyGEN Biotech (Nanjing, China).

MCP was prepared as previously reported [50].

### Western blot analysis

Cells were harvested and treated with lysis buffer (50 mM Tris/acetate, pH 7.4, 0.5% Triton X-100, 150 mM sodium chloride, 0.1 mM PMSF, Roche complete protease inhibitor cocktail and 2 mM Na_3_VO_4_) for 30 min on ice and then centrifuged at 13,000 g for 15 min at 4°C. The supernatant was collected and quantitated. Thirty micrograms of protein lysates from each sample were separated on a 12% SDS–PAGE and transferred onto PVDF membranes (Roche). Membranes were then blocked with 5% nonfat dry milk in 1× TBST (PBS containing 0.05% Tween 20) for 1 h, incubated with appropriate primary antibodies (1:1000) for 1 h at room temperature, followed by HRP-conjugated goat anti-rabbit/mouse IgG (1:5000) after washing thrice with 1× TBST. All the protein blots were developed by ECL Plus Western Blotting Detection Kit (GE Healthcare, Milwaukee, WI, USA) and quantified using the automatic chemiluminescence imaging analysis system (Tanon 5500, Shanghai, China). The antibodies against caspase-3 (cat. no. 9662S), caspase-9 (9502S), ERK (9012S), p-ERK (9011S), JNK (9252S), p-JNK (9251S), p38 (9212S), p-p38 (9215S), p-PKC (2060S), p-PI3K (4228S), Akt (4691S), p-Akt (T308) (13038S), p-Akt (S473) (9271S), c-Raf (9422S), p-c-Raf (9427S) and CD71 (13208S) were purchased from Cell Signaling Technology (Beverly, MA, USA). The antibodies against CD45 (610265) and CD29 (610467) were from BD PharMingen (San Diego, CA, USA). The β-actin antibody (cat. no. 612657) was from BD Biosciences (San Jose, CA, USA).

### Flow cytometry analysis

To analyze apoptosis, cells were harvested after treatments, washed thrice with PBS, re-suspended in the binding buffer (PI/FITC-Annexin V Apoptosis Detection Kit (KGA 107) from KeyGEN Biotech (Nanjing, China)), and stained with PI/FITC-AnnexinV for 30 min according to the manufacturer's instructions. Flow cytometry was perfomed in the Beckman Coulter Epics XL^TM^ flow cytometer with excitation at 488 nm and emission at 530 nm for FITC-Annexin V and 625 nm for PI, respectively. For each sample, 10,000 cells were analyzed using Expo32 software.

For the ROS measurement, cells were harvested and washed twice with PBS, and resuspended in 200 μl serum free RPMI 1640 medium. Then, the cells were stained with 10 μM H_2_DCFDA for 20 min at 37°C in the dark, washed thrice with serum free RPMI 1640 medium and analyzed by FACS with excitation at 488 nm and emission at 530 nm. For each sample, at least 10,000 cells were analyzed using the Expo32 software. For data in Figure 4C and Figure 7D, ROS measurements were determined by a BD Accuri^TM^ C6 flow cytometer (Ex=488nm, Em=530nm) and analyzed with BD Accuri^TM^ C6 software.

### Pull down assay

Twenty micrograms of recombinant His-Gal-3 or His-G69-250 was incubated with 20 μL Ni-NTA beads for 2 h at 4°C with constant agitation by rotation. Then, the beads were washed thrice in PBS and further incubated with Jurkat cell lysate (5×10^6^ cells) for another 2 h at 4°C. Then the beads were collected, washed, boiled in SDS-PAGE sample buffer and analyzed by western blotting.

### siRNA transfection

Jurkat cells were transfected with 75 nM of each siRNA using HiPerFect transfection reagent (Qiagen, GmbH, Hilden, Germany) according to the manufacturer's instructions. The siRNA sequences were as follows: 5′-UUCUCCGAACGUGUCACGU-3′ for the negative control (NC); 5′-GCUGCAAUGUGUCAUUUCA-3′ for CD45; 5′-CAUUGGAGAUGAGGUUCAA-3′ for CD29; and 5′-AACUUCAAGGUUUCUGCCAGC-3′ for CD71.

## SUPPLEMENTARY FIGURE


