# Supplementary material for: The N-terminal tail coordinates with carbohydrate recognition domain to mediate galectin-3 induced apoptosis in T cells
Source: Oncotarget. 2017 May 10;8(30):49824–38. doi: 10.18632/oncotarget.17760 (PMC5564810; doi:10.18632/oncotarget.17760)
Supplement: Supplementary file 1 [file oncotarget-08-49824-s001.pdf]

The N-terminal tail coordinates with carbohydrate recognition domain to mediate galectin-3 induced apoptosis in T cells

SUPPLEMENTARY MATERIALS

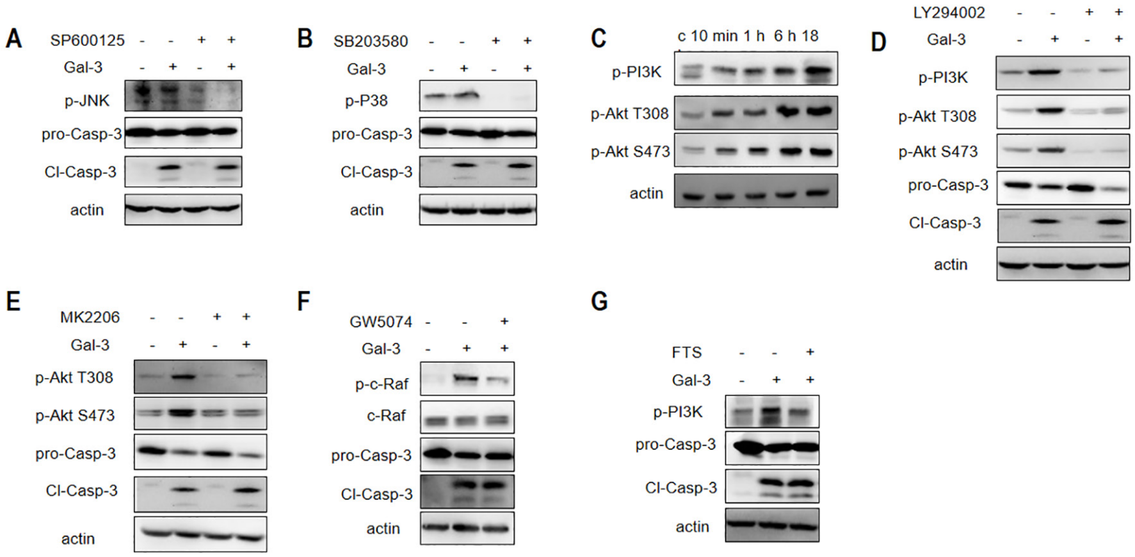

Supplementary Figure 1: Effect of different inhibitors on Gal-3-triggered T cell apoptosis. (A-B) Jurkat cells were incubated with Gal-3 in the presence or absence of SP600125 or SB203580 for 18 h, followed by western blot analysis of p-JNK (A) or p-P38 (B) and cleaved caspase-3. **(C)** Jurkat cells were incubated with Gal-3 and p-PI3K, p-Akt (T308) and p-Akt (S473) were assessed by western blotting. **(D-G)** Jurkat cells were incubated with Gal-3 in the absence or presence of LY294002, MK2206, FTS or GW5074 for 18 h. Western blotting analysis of p-PI3K (D and G), p-Akt (T308), p-Akt (S473) (D and E), p-c-Raf, c-Raf (F) and cleaved caspase-3 (D-G) was determined.
